# Synthesis and Characterization of Biopolymeric Chitosan Derived from Land Snail Shells and Its Potential for Pb^2+^ Removal from Aqueous Solution

**DOI:** 10.3390/ma8125482

**Published:** 2015-12-10

**Authors:** Joshua N. Edokpayi, John O. Odiyo, Elizabeth O. Popoola, Oluwagbemiga S. Alayande, Titus A. M. Msagati

**Affiliations:** 1 Department of Hydrology and Water Resources, University of Venda, Private Bag X5050, Thohoyandou 0950, South Africa; john.odiyo@univen.ac.za; 2 Department of Chemical Sciences, Yaba College of Technology, P.M.B. 2011, Yaba, Lagos 101212, Nigeria; seunliz27@yahoo.com; 3 Centre for Energy Research and Development, Obafemi Awolowo University, P.M.B. 13, Ile Ife 220, Nigeria; gbengaalayande@googlemail.com; 4 Nanotechnology and Water Sustainability Research Unit, College of Science, Engineering and Technology, Florida Science Campus, University of South Africa, 1710 Roodepoort, Johannesburg 2000, South Africa; msagatam@unisa.ac.za

**Keywords:** adsorption, chitosan, kinetics, snail shells

## Abstract

Pb^2+^ is considered to be a very toxic pollutant in the aquatic environmental media. Biopolymeric chitosan synthesized from snail shell has been studied for its potential to remove heavy metals from aqueous solution. The experiments were conducted in the range of 1–50 mg/L initial Pb^2+^ concentration at 298 K. The effects of pH, adsorbent dosage and contact time on the adsorptive property of the adsorbent were investigated and optimized. The derived chitosan was characterized using Fourier transform infrared spectrometer (FT-IR) and X-ray florescence (XRF). The experimental data obtained were analysed using the Langmuir and Freundlich adsorption isotherm models. The Langmuir model and pseudo second order kinetic model suitably described the adsorption and kinetics of the process with regression coefficients of 0.99 and 1.00, respectively. Sodium hydroxide was a better desorbing agent than hydrochloric acid and de-ionized water. From the results obtained, it is concluded that synthesized biopolymers from land snail shells has the potential for the removal of Pb^2+^ from aqueous solutions.

## 1. Introduction

Heavy metals are a group of pollutants occurring naturally in the earth crust. The pollution of environmental matrices has been of global concern due to their relative persistence in the environment [1]. Rapid industrialization coupled with population growth and increased urbanization has led to an increase in heavy metal concentrations [2,3]. Pb^2+^ is known to be part of the composition of effluents that are found in both domestic and industrial sources. Apart from the background concentration of Pb^2+^ in water, its presence can be attributed to the corrosion of lead pipes, the use of gasoline containing lead and the release of domestic and industrial effluents [4]. The toxic effect of Pb^2+^ is well known and several governmental and non-governmental agencies like the World Health Organization (WHO), United States Environmental Protection Agency (US EPA) and the South African Department of Water and Sanitation have established threshold values on the concentration of Pb^2+^ in drinking water and wastewaters [5]. Pregnant women and children are the most vulnerable to Pb^2+^ contamination even at low concentrations if exposed for a very long period of time [6,7].

Some conventional methods have been reported for the removal of heavy metals from water and wastewater. These include chemical precipitation [2,8,9], ion exchange [2], liquid membrane extraction [10], chemical coagulation and flocculation [11]. These methods are ineffective at low metal concentrations and generate a lot of sludge that cause disposal problems [9]. Other methods like adsorption using commercial activated carbon [12,13], microfiltration [14], reverse osmosis [9,14] and membrane techniques [9] are effective, but the cost of installation and maintenance are expensive which limit their use. These drawbacks have led to a continuous search for a cheap, renewable and more economic method for the removal of unwanted metals from aqueous solution.

Chitosan is an important natural biopolymer usually derived from the deacetylation of chitin from the shells of shrimp, crab, and other arthropods and has been widely used in pharmaceutical industries for drug delivery [15]. Sewvandi and Adikary [16] studied the removal of Cr^6+^ from wastewater using chitosan derived from shrimp shells. Similarly, Mohanasrivinidasa *et al.* [17] investigated the removal efficiencies of metals and antimicrobial activity of chitosan derived from shrimp shells. Inference from their studies suggests the potential use of crustacean shells as adsorbents for heavy metal remediation [17]. Jatto *et al.* [18] investigated the coagulative property of land snail shell in reducing water quality parameters of wastewater from food industries; their studies showed a reduction in the concentrations of nitrates, sulphates and chemical oxygen demand.

Land snails are widely consumed in different parts of the world. The shells are often disposed indiscriminately and therefore constitute a nuisance to the environment. Several degrees of injuries to children caused by discarded snail shells have been observed. This study explores the potential of preparing chitosan (a natural, biodegradable, biocompatible, bio-adhesive polymer) from the shells of land snails and using it as an adsorbent for the removal of Pb^2+^ from aqueous solution.

## 2. Experimental

### 2.1. Materials

Discarded land snail shells were collected from a local market at Ile-Ife, Nigeria. The shells were thoroughly washed to remove leaves, sand, dirt and other impurities. They were dried at 100 °C and pulverized into fine powder. The powder was sieved using an analytical sieve of 250 micron. Analytical grade chemicals were used in this study. Hydrochloric acid (32%) and sodium hydroxide were supplied by Sigma Aldrich (Johannesburg, South Africa) while 1000 mg/L of Pb^2+^ was supplied from Merck (pty) Ltd. (Johannesburg, South Africa).

### 2.2. Preparation of Chitosan

The method reported by Mohanasrinivasan *et al.* [17] was employed with slight modification. Eighty grams of the powder was weighed into a conical flask and 100 mL of 4% NaOH was added. The mixture was boiled and stirred at 100 °C for 2 h in a water bath. After boiling and stirring, it was filtered and washed with distilled water. Red litmus was used to check if the base was completely washed away. After washing, the mixture was filtered and the residue was scraped gently into the petri dishes and then dried in the oven at 100 °C for 3 h.

After deproteinization, the weight of the sample was 62 g. Thirty milliliters of 5% 1 M HCl was added to the deproteinized sample. The mixture was boiled and stirred for 45 min at 100 °C in a water bath. Subsequent washing was done with distilled water followed by filtration. The mixture was examined with blue litmus to check the acidity of the mixture. The residue (chitin) obtained from above was scarped into the petri dish and dried in the oven at 100 °C for 2 h.

Deacetylation reaction was used to convert chitin to chitosan according to a revised procedure of Coughlin *et al.* [19]. Briefly, the isolated chitin was soaked in 510 mL of 50% NaOH (weighing 50 g of NaOH pellets and dissolved in 100 mL of distilled water), boiled at 100 °C for 2 h in water bath and cooled for 30 min at room temperature. The mixture was placed on a magnetic stirrer at 30 °C for 4 h, filtered, washed and examined with red litmus to check if the base was completely washed away. The mixture was filtered to retain the solid matter which is chitosan. The chitosan was oven dried at 90 °C for 24 h.

### 2.3. Characterization of the Synthesized Chitosan

Elemental studies were performed with a Rigaku ZSX Primus II X-ray Fluorescence spectrometer (USA). Fourier transform infrared (FT-IR) spectra of the synthesized chitosan from snail shells were obtained using a Perkin Elmer 100 FT-IR (Waltham, MA, USA) with accessories. The sample pellets were prepared by using a KBr press (Spectra Lab, Mumbai, India). The spectra was scanned over the wave number range of 4500 to 400 cm^−1^. The synthesized chitosan were coated with a thin layer of carbon and the surface morphology was analyzed using a scanning electron microscope (SEM) (TESCAN, VEGA 3 SBU, Brno, Czech) and irradiated with a beam of electrons at 20 kV. Surface area and pore width were determined by N_2_ gas Brunauer-Emmett-Teller method of analysis using a Micrometrics Chemisorption ASAP 2020 supplied by Norcross, GA, USA. A Perkin Elmer thermal analyzer (Waltham, MA, USA) was used for thermal degradation studies of the adsorbent.

### 2.4. Adsorption and Kinetics

A Stuart reciprocal shaker with a speed of 250 rpm and a temperature of 298 K was employed for all the experiments in this study. 0.1 M NaOH and 0.1 M HCl were used to adjust the pH of the solution to the desired value. The effect of adsorbent dosage was studied by varying the mass of the synthesized chitosan in the range of 0.05 to 2.0 g using a 40 mL of 10.7 mg/L Pb^2+^ solution. Optimum pH values and contact time were obtained by varying the pH value from 2 to 12 while the equilibration time was varied from 5 to 120 min. The effect of initial Pb^2+^ concentration was performed using different Pb^2+^ concentrations between 1–50 mg/L. In all cases, after equilibration, the solution was centrifuged for 3 min and filtered using a 0.45 µm filter membrane. The filtrates were subsequently analysed using Atomic Absorption Spectrometer.

### 2.5. Desorption Studies

Regeneration of the adsorbent was performed using de-ionized water, 0.1 M HCl and 0.1 M NaOH. One gram of the adsorbent was agitated with 10.7 mg/L of Pb^2+^ on a Stuart reciprocal mechanical shaker for 60 min at 250 rpm. After equilibration, the adsorbent was separated by centrifugation and subsequently rinsed three times using de-ionized water to remove any unadsorbed Pb^2+^ on the surface. The already used adsorbent was agitated with the three desorbing agents at the same experimental conditions. The filtrates were analyzed using flame Atomic Absorption Spectrometer.

## 3. Results and Discussion

### 3.1. Characterization of the Synthesized Chitosan

**Figure 1:**
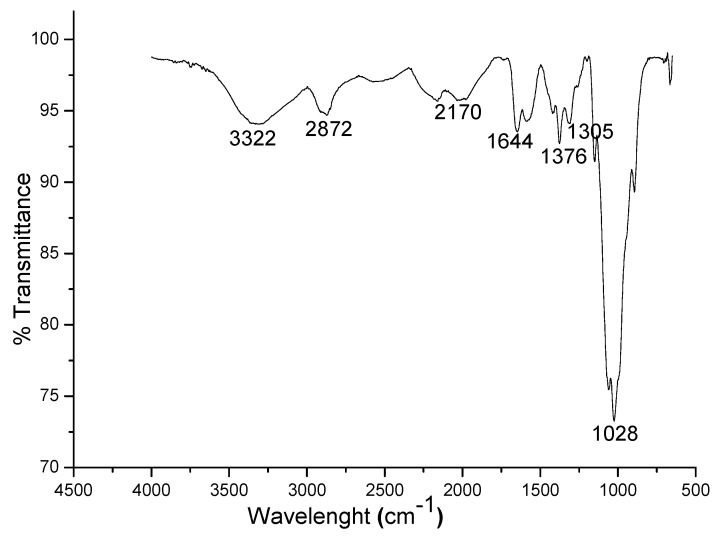
Infrared spectra of the adsorbent.

The band observed at 3322 cm^−1^ (Figure 1) can be attributed to –NH_2_ or –OH groups stretching vibration [20]. The peak observed at 2915 cm^−1^ indicated alkane –C-H stretching vibration or –OH stretch of carboxylic acids, while that of 2524 cm^−1^ can be assigned to –C≡C stretching vibration of alkynes [21]. The characteristic –NH band of chitosan was observed at 1644 cm^−1^ [22]. The bands observed at 1376 cm^−1^ and 1305 cm^−1^ can be attributed to –NO stretch of nitrogen containing compounds and –CO stretch of carbonyl compounds, respectively [22]. The observed peak at 1028 cm^−1^ is assigned to –CN stretch of aliphatic amines [22]. The elemental analysis of the synthesized chitosan (Table 1) showed calcium as the major element making up to 98.2%.

**Table 1:** Chemical analysis of synthesized chitosan.

Component	wt % Composition
Na_2_O	0.58
MgO	0.03
Al_2_O_3_	0.15
SiO_2_	0.34
P_2_O_5_	0.07
SO_3_	0.10
Cl	0.02
K_2_O	0.04
CaO	98.20
Fe_2_O_3_	0.10
SrO	0.40

**Figure 2:**
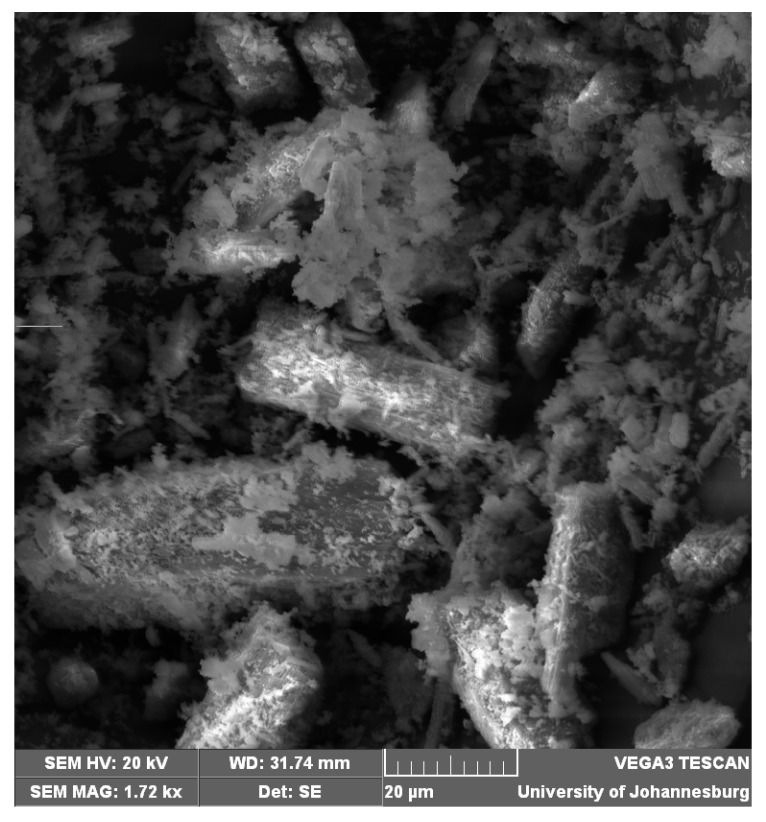
SEM micrograph of the adsorbent.

SEM analysis is a useful tool for investigation of the surface area and structure morphologies of various adsorbents [23]. The SEM micrograph of the synthesized chitosan (Figure 2) shows a rough surface with significant pores and remarkable irregularities suitable for the adsorption of metals. BET surface area of 1.7998 m^2^/g was determined for the adsorbent. The pore volume (1.492 × 10^−2^ cm^3^/g) and average pore diameter (21.66 nm) determined is characteristic of a mesoporous material [24,25].

**Figure 3:**
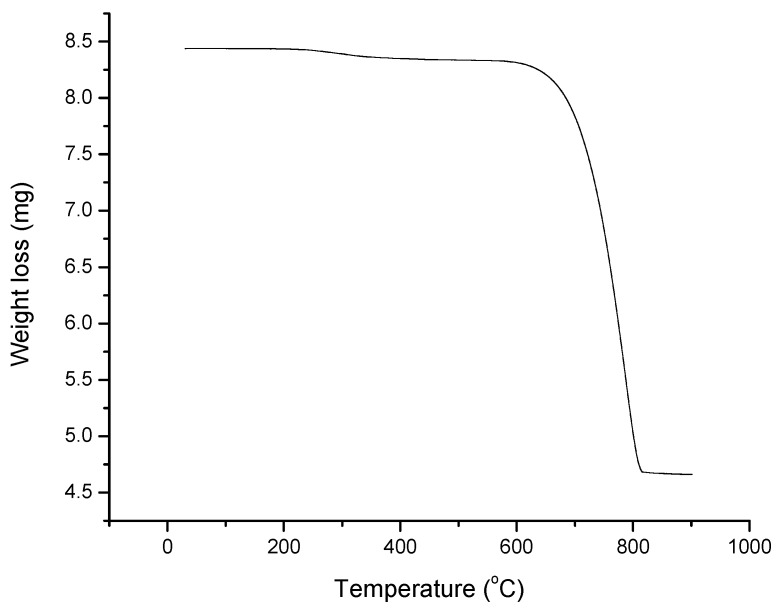
TGA profile of synthesized Chitosan.

The synthesized chitosan (8.5 mg) was heated from 30 °C to 900 °C at 20 °C·min^−1^ using a thermogravimentary analyzer. The biosorbent was stable up to 610 °C (Figure 3) although a little loss in mass was recorded between 100 and 200 °C, which can be attributed to loss of bound water molecules in the samples. The sample degraded at temperatures between 610 and 813 °C, which accounted for about 43% of the initial biosorbent. A slight degradation was observed after this point.

### 3.2. Effects of Experimental Conditions on Adsorption Efficiency

**Figure 4:**
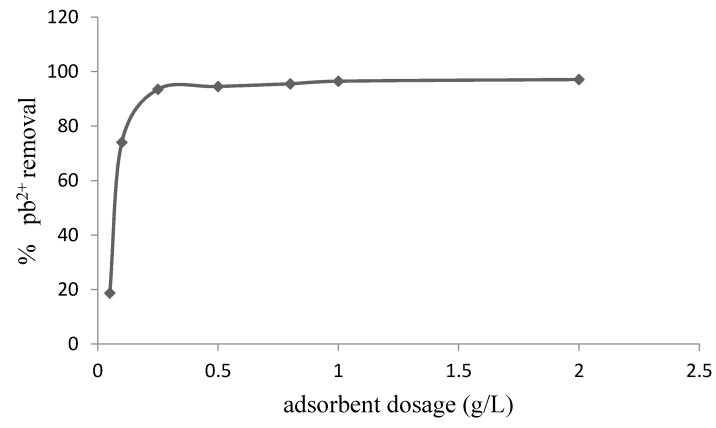
Variation of percent Pb^2+^ removal with adsorbent dosage (initial concentration = 10.7 mg/L, contact time = 40 min, T = 298 K, pH = 4, shaking speed = 250 rpm).

The percentage uptake of Pb^2+^ by the synthesized chitosan was found to increase with increasing quantity of the adsorbent from 0.05 to 2.0 g/L (Figure 4). This finding can be attributed to the increase in binding sites on the adsorbent as the number of available binding sites is expected to increase with increased quantity of the adsorbent [25,26]. However, the amount of Pb^2+^ adsorbed per unit mass of the adsorbent decreases at increasing dosage due to overlapping of the adsorption sites by Pb^2+^. From the experimental data obtained, 1.0 g/L of the synthesized chitosan was found to achieve maximum adsorption of Pb^2+^.

**Figure 5:**
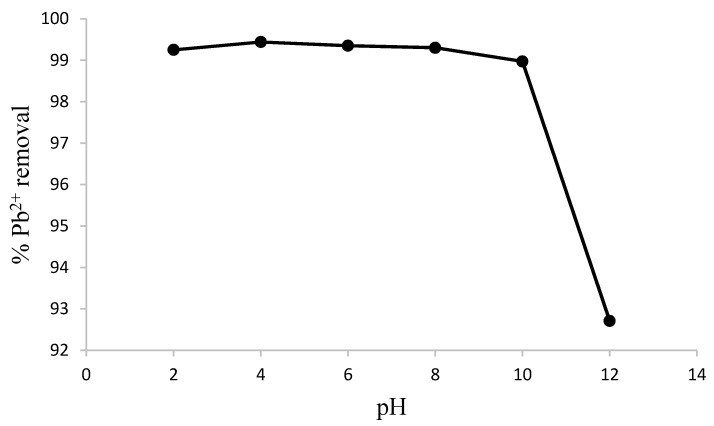
Variation of percent Pb^2+^ ion removal with change in pH value (initial concentration = 10.7 mg/L, contact time = 40 min, T = 298 K, shaking speed = 250 rpm, adsorbent dosage = 1.0 g).

The dependence of adsorption of Pb^2+^ was nearly constant between pH values of 2 to 10, although a slight increase in the uptake of Pb^2+^ was recorded between pH 2 and 4. The optimum pH value for this study is four (Figure 5). This can be attributed to electrostatic balance between the protonated amine sites and Pb^2+^ [27,28]. There was a decrease in the uptake of Pb^2+^ by the synthesized chitosan at pH > 10. The cause for the phenomenon could be due to reduced solubility and precipitation of Pb^2+^ under alkaline condition [26]. Pb^2+^ is soluble in pH < 4 but will precipitate out of solution under neutral and alkaline conditions. The mechanism of the interaction based on the optimum pH is purely by adsorption while that of alkaline pH can be regarded as a mixture of adsorption/precipitation [27,28].

**Figure 6:**
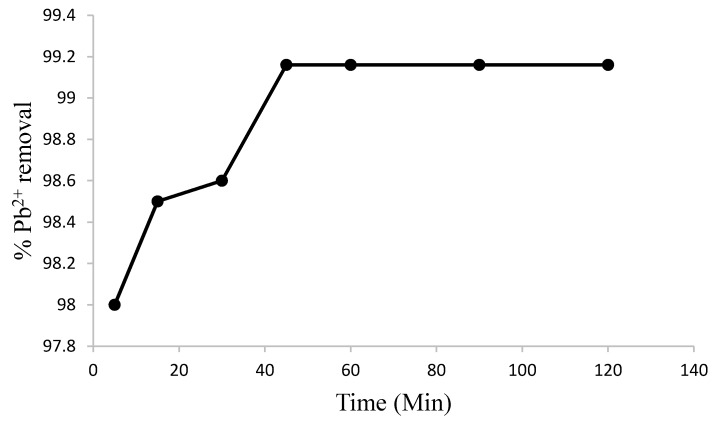
Variation of percent Pb^2+^ ion removal with contact time (initial concentration = 10.7 mg/L, T = 298 K, pH = 4, shaking speed = 250 rpm, adsorbent dosage =1.0 g).

There was a rapid uptake of Pb^2+^ by the synthesized chitosan within 5 min of equilibration (98%) which is due to the availability of vacant binding sites on the surface of the adsorbent. A gradual increase in the uptake of Pb^2+^ was further recorded up to 40 min of equilibration (99%) due to the filling of the remaining binding sites on the adsorbent. Beyond this time (Figure 6), no significant increase in the adsorption capacity of Pb^2+^ was determined, indicating that equilibrium has been reached. This phenomenon can be explained as due to repulsive forces between Pb^2+^ on the surface of the adsorbent and Pb^2+^ in the aqueous phase [25]. Similar optimum time for Pb^2+^ sorption has been reported by Hikmat *et al.* [29], although Santi *et al.* [30] and Tahiruddin and Ab Rahman [31] reported an optimum time of 50 min and 30 min for the adsorption of Pb^2+^ onto activated carbon from rice husk and peanut shells, respectively.

### 3.3. Adsorption Studies

The adsorption isotherm is an extremely important tool for understanding the distribution of the adsorbate on the adsorbent surface at equilibrium [32,33]. Langmuir and Freundlich isotherm models were used to describe the equilibrium data obtained. Langmuir isotherm applies to the monolayer adsorption process while the Freundlich isotherm is a semi empirical equation based on the adsorption process that occurs on heterogeneous surfaces [34,35]. The linearized equation of Langmuir and Freundlich isotherm models are:

(1) $$\frac{1}{q_{e}}=\frac{1}{q_{\max}}+\left(\frac{1}{bq_{\max}}\right)\frac{1}{C_{e}}$$

(2) $$\log q_{e}=\log K_{f}+\left(\frac{1}{n}\right)\log C_{e}$$

**Figure 7:**
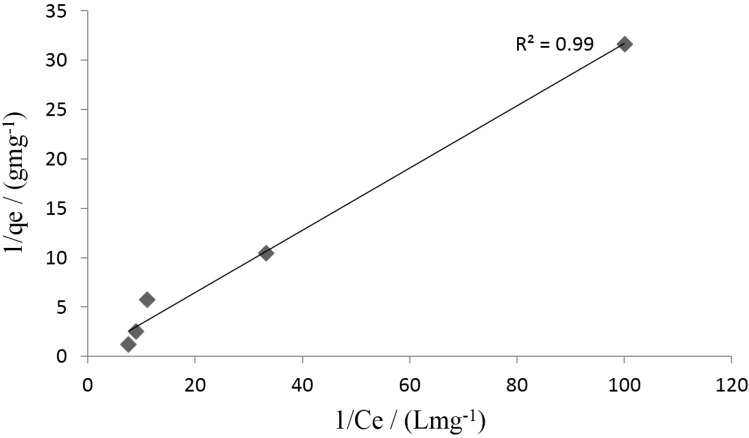
Langmuir plot for Pb^2+^ adsorption onto synthesized chitosan.

A plot of 1/*q_e versus_* 1/C_e_ gave a straight line with a regression coefficient (*R^2^*) of 0.99 (Figure 7) with 1/*q*_max_ as intercept and 1/*bq*_max_ as slope. Langmuir isotherm can also be expressed in terms of a dimensionless constant called separation factor R_L_ [34];

(3) $$R_{L}=\frac{1}{\left(1+bC_{O}\right)}$$

where *C_o_* is the initial metal concentration (mg/L) and b the Langmuir constant (L/mg). *R_L_* > 1 indicates an unfavourable monolayer adsorption process, *R_L_* = 1 linear, 0 < *R_L_* < 1 favourable and *R_L_* = 0 irreversible [24,36]. The result obtained from this study has an R_L_ value between zero and one, indicating a favourable adsorption process. This implies that the chemisorption process duly explains the adsorption process.

**Figure 8:**
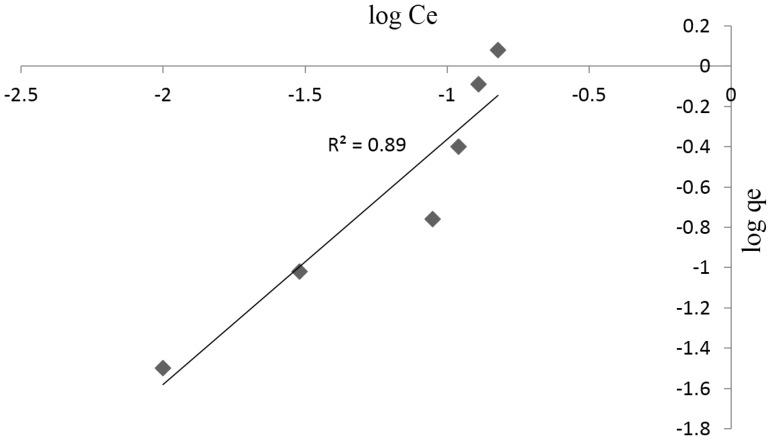
Freundlich plot for Pb^2+^ adsorption onto synthesized chitosan.

Figure 8 shows the plot of log q_e_ against log Ce, which also gave a straight line with a linearized coefficient of 0.89, implying that the adsorption process could also be controlled by physisorption as a result of weak van der Waals forces between the surface of adsorbent and adsorbate. Based on the correlation coefficients obtained from the Langmuir and Freundlich isotherm models, it can be deduced that the adsorption process is more favored by the Langmuir isotherm model. This is consistent with other results reported in literature [37,38].

### 3.4. Kinetic Studies

**Figure 9:**
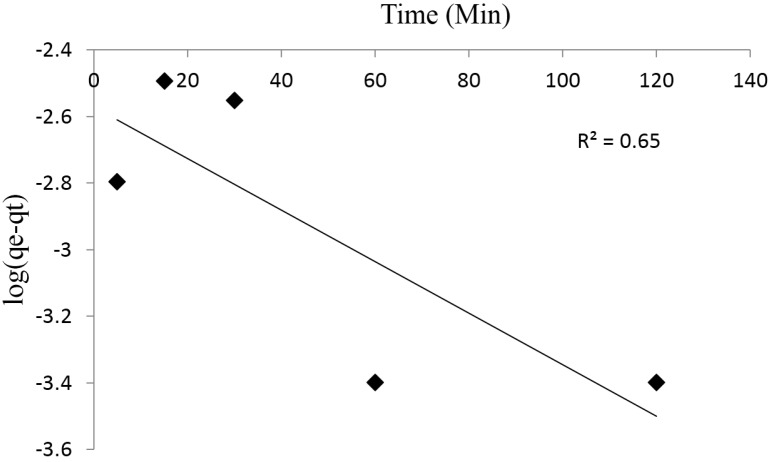
Pseudo first order kinetics for Pb^2+^ adsorption onto synthesized chitosan.

**Figure 10:**
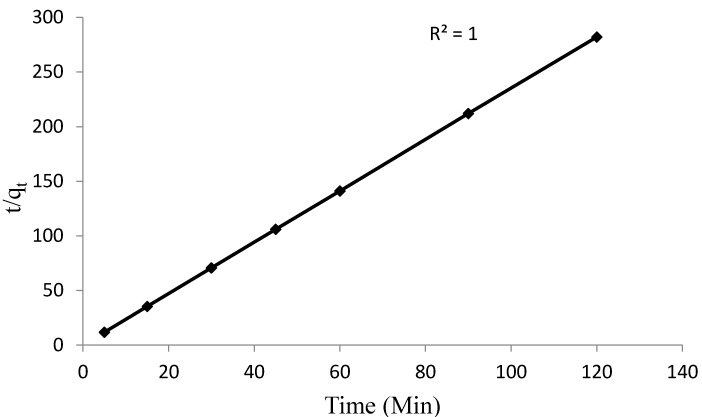
Pseudo second order kinetics for Pb^2+^adsorption onto synthesized chitosan.

To explain the kinetics of the adsorption process, the experimental data were subjected to the pseudo first order and pseudo second order kinetic models. Equations (4) and (5) show the linearized forms of the pseudo first and pseudo second order kinetic models, respectively.

(4) $$\log(q_{e}-q_{t})=\log q_{e}-(\frac{k_{1}}{2.303})$$

(5) $$\frac{t}{q_{t}}=(\frac{1}{k_{2}q_{e}^{2}})+\frac{t}{q_{e}}$$

where *q_e_* and *q_t_* are the amounts of Pb^2+^ adsorbed at equilibrium and at time *t*, *k*_1_ and *k*_2_ are the rate constants of the pseudo first and pseudo second order model. The correlation coefficients obtained from the plots of the pseudo first order and pseudo second order models are 0.65 and 1.0, respectively (Figure 9 and Figure 10). The pseudo second order best describes the kinetics of the adsorption process which corroborate with the findings of Ahmad *et al.* [34], Moyo and Chikazaza [39] and Chen *et al.* [40] in the adsorption of Pb^2+^ by macrocylic calyx[4]naphthalene, acid treated maize tassels and thiacalix[4]arene composites, respectively.

### 3.5. Desorption

De-ionized water and 0.1 M HCl were very poor desorbing agents, removing less than 5% of the adsorbed Pb^2+^ from the surface of the adsorbent while 0.1 M NaOH was a better desorbing agent with about 30% removal. This also suggests the formation of a bond between the surface of the adsorbent and Pb^2+^.

## 4. Conclusions

This study has shown that synthesized chitosan from land snail shells can be employed for the adsorption of Pb^2+^ from aqueous solution and wastewater. The mechanism of the adsorption of Pb^2+^ onto the adsorbent is believed to be through the adsorption of the metal onto the amine functional group of the adsorbent. The equilibrium data obtained fit better into the Langmuir isotherm model than the Freundlich isotherm model, implying a chemisorption mechanism.

## References

[1] Hernandez-Montoya V., Perez-Cruz M.A., Mendoza-Casillo D.I., Moreno-Virgen M.R., Bonilla-Petriciolet A. (2013). Competitive adsorption of dyes and heavy metals on zeolitic structure. J. Environ. Manag..

[2] Das N., Karthiica P., Vimala R., Vinidhini V. (2008). Use of natural products as biosorbent of heavy metals, An overview. Nat. Prod. Radiance.

[3] Cataldo S., Muratore N., Orecchio S., Pettignano A. (2015). Enhancement of adsorption ability of calcium alginate gel beads towards Pd(II) ion. A kinetic and equilibrium study on hybrid Laponite and Montmorillonite-alginate gel beads. Appl. Clay Sci..

[4] Koh L.L., Wong M.K., Gan L.M. (1991). Factors affecting the leaching of lead from UPVC pipes. Environ. Monit. Asses..

[5] Sublet R., Simonnot M., Boireau A., Sardin M. (2003). Selection of an adsorbent for lead removal from drinking water by a point-of-use treatment device. Water Res..

[6] Minnesota Department of Health (2010). Point-of-Use Water Treatment Units for Lead Reduction. 141–0717. http://www.health.state.mn.us/divs/eh/water/factsheet/com/poulead.html.

[7] Edokpayi J.N., Odiyo J.O., Olasoji S.O. (2014). Assessment of heavy metal contamination of Dzindi River, in Limpopo Province, South Africa. Int. J. Nat. Sci. Res..

[8] Zvinowanda C.M., Okonkwo J.O., Agyei N.M., Shabalala P.N. (2009). Physicochemical characterization of maize tassel as an adsorbent. I. surface texture, microstructure and thermal stability. J. Appl. Polym. Sci..

[9] Mihajlovic M.T., Lazarevic S.S., Jankovic-Castvan I.M., Kovac J., Jokic B.M., Janackovic D.T., Petrovic R.D. (2015). Kinetics, thermodynamics, and structural investigations on the removal of Pb^2+^, Cd^2+^, and Zn^2+^ from multicomponent solutions onto natural and Fe(III)-modified zeolites. Clean Technol. Environ. Policy.

[10] Rorrer G., Meyers R.A. (1998). Heavy metal ions. Removal from Wastewater: Encyclopaedia of Environmental Analysis and Remediation.

[11] Johnson P.D., Padmanabhan G.P., Ohlinger K.N., Ritchie S., Teuber L., Kirby J. (2008). Enhanced removal of heavy metals in primary treatment using coagulation and flocculation. Water Environ. Res..

[12] Freeman H.F. (1989). Standard Handbook of Hazardous Waste Treatment and Disposal.

[13] Cheremisinoff N.P. (2002). Handbook of Water and Wastewater Treatment Technologies.

[14] Geselbarcht J. Micro filtration/reverse osmosis pilot trials for Livermore, California, advanced water reclamation. Proceedings of the AWWA 1996 Water reuse conference.

[15] Ilium L. (1998). Chitosan and its use as a pharmaceutical excipient. Pharm. Res..

[16] Sewvandi G.A., Adikary S.U. (2011). Removal of Heavy Metals from Wastewater Using Chitosan. Society for Social Management Systems Internet Journal.

[17] Mohanasrinivasan V., Mishra M., Paliwal J.S., Singh S.K., Selvarajan E., Suganthi V., Devi C.S. (2014). Studies on heavy metal removal efficiency and antibacterial activity of chitosan prepared from shrimp shell waste. 3 Biotech..

[18] Jatto E.O., Asia I.O., Egbon E.E., Otutu J.O., Chukwuedo M.E., Ewansiha C.J. (2010). Treatment of waste water from food industry using snail shell. Acaedmia Arena.

[19] Coughlin R.W., Deshaies M.R., Davis E.M. (1990). Preparation of chitosan for heavy metal removal. Environ. Prog..

[20] Bhumkar D.R., Pokharkar V.B. (2006). Studies on effect of pH on cross-linking of chitosan with sodium tripolyphosphate: A technical note. APS PharmSciTech.

[21] Ray M., Anis K.P.A., Banthia A.K. (2010). Development and characterization of chitosan based polymeric hydrogel membranes. Des. Monomers Polym..

[22] Coates J., Meyers R.A. (2000). Interpretation of Infrared Spectra, a practical approach. Encyclopedia of Analytical Chemistry.

[23] Özbay İ., Özdemir U., Özbay B., Veli S. (2013). Kinetic, thermodynamic, and equilibrium studies for adsorption of azo reactive dye onto a novel waste adsorbent: Charcoal ash. Desalin. Water Treat..

[24] Edokpayi J.N., Odiyo J.O., Msagati T.A.M., Popoola E.O. (2015). A Novel Approach for the removal of lead(II) ion from wastewater using mucilaginous leaves of diceriocaryum eriocarpum plant. Sustainability.

[25] Lingamdinne L.P., Koduru J.R., Jyothi R.K., Chang Y., Yang J. (2015). Factors affect on bioremediation of Co(II) and Pb(II) onto Lonicera japonica flowers powder. Desalin. Water Treat..

[26] Koduru J.R., Chang Y., Yang J., Kim I. (2013). Iron oxide impregnated morus alba l. fruit peel for biosorption of Co(II): Biosorption properties and mechanism. Sci. World J..

[27] Dambies L., Guimon C., Yiacoumi S., Guiba E. (2001). Characterization of metal ion interactions with chitosan by X-ray photoelectron spectroscopy. Coll. Surf. A Physicochem. Eng. Asp..

[28] Guibal E. (2004). Interactions of metal ions with chitosan-based sorbents: A review. Sep. Puri. Technol..

[29] Hikmat N.A., Qassim B.B., Khethi M.T. (2014). Thermodynamic and kinetic studies of lead adsorption from aqueous solution onto petiole and fibre of palm tree. Am. J. Chem..

[30] Raya I., Zakir M. (2014). The adsorption of Pb (II) ions on activated carbon from rice husk, irradiated by ultrasonic waves: Kinetic and thermodynamics studies. J. Nat. Sci. Res..

[31] Tahiruddin N.S.M., Ab Rahman S.Z. (2013). Adsorption of lead in aqueous solution by a mixture of activated charcoal and peanut shell. World J. Sci. Technol. Res..

[32] Kannamba B., Reddy K.L., AppaRao B.V. (2010). Removal of Cu (II) from aqueous solutions using chemically modified chitosan. J. Hazard. Mater..

[33] Rabelo R.B., Vieira R.S., Luna F.M.T., Guibal E., Beppu M.M. (2012). Adsorption of copper(II) and mercury(II) ions onto chemically-modified chitosan membranes: Equilibrium and kinetic properties. Adsorpt. Sci. Technol..

[34] Ahmad R., Kumar R., Laskar M.A. (2013). Adsorptive removal of Pb^2+^ form aqueous solution by macrocyclic calyx[4]naphthalene: Kinetic, thermodynamic, and isotherm analysis. Environ. Sci. Pollut. Res..

[35] Gupta V K., Ali I. (2008). Removal of endosulfan and methoxychlor from water on carbon slurry. Environ. Sci. Technol..

[36] Gupta V.K., Gupta M., Shamar S. (2001). Process development for the removal of lead and chromium from aqueous solutions using red mud—An aluminium industry waste. Water Res..

[37] Asandei D., Bulgariu L., Bobu E. (2009). Lead(II) removal from aqueous solutions by adsorption onto chitosan. Cellul. Chem. Technol..

[38] Ng J.C.Y., Cheung W.H., Mckay G. (2002). Equilibrium studies of the sorption of Cu(II) ions onto chitosan. J. Colloid Interface Sci..

[39] Moyo M., Chikazaza L. (2013). Bioremediation of lead(II) from polluted wastewaters employing sulphuric acid treated maize tassel biomass. Am. J. Anal. Chem..

[40] Chen D., Hu B., He M., Haung C. (2010). Micro-column preconcentration/separation using thiacalix[4]arene tetracarboxylate derivative modified mesoporous TiO_2_ as packing materials on-line coupled to inductively coupled plasma optical emission spectrometry for the determination of trace heavy metals in environmental water samples. Microchem. J..

